# Preparative Isolation of Two Prenylated Biflavonoids from the Roots and Rhizomes of *Sinopodophyllum emodi* by Sephadex LH-20 Column and High-Speed Counter-Current Chromatography

**DOI:** 10.3390/molecules21010010

**Published:** 2015-12-23

**Authors:** Yan-Jun Sun, Li-Xin Pei, Kai-Bo Wang, Yin-Shi Sun, Jun-Min Wang, Yan-Li Zhang, Mei-Ling Gao, Bao-Yu Ji

**Affiliations:** 1 Collaborative Innovation Center for Respiratory Disease Diagnosis and Treatment & Chinese Medicine Development of Henan Province, Henan University of Traditional Chinese Medicine, Zhengzhou 450046, Henan, China; xlpjby@sina.com (L.-X.P.); wjmhnzz@163.com (J.-M.W.); zyl2013hnzy@163.com (Y.-L.Z.); gaoxiaomei6266@126.com (M.-L.G.); ys20052@sina.com (B.-Y.J.); 2 School of Pharmacy, Henan University of Traditional Chinese Medicine, Zhengzhou 450046, Henan, China; 3 Key Laboratory of Structure-Based Drug Design & Discovery, Ministry of Education, Shenyang Pharmaceutical University, Shenyang 110016, Liaoning, China; wangkaibo2014@163.com; 4 School of Traditional Chinese Materia Medica, Shenyang Pharmaceutical University, Shenyang 110016, Liaoning, China; 5 Institute of Special Animal and Plant Sciences, Chinese Academy of Agricultural Sciences, Changchun 130112, Jilin, China

**Keywords:** *Sinopodophyllum emodi*, prenylated biflavonoid, Sephadex LH-20 column chromatography, high-speed counter-current chromatography, cytotoxic activity

## Abstract

Two prenylated biflavonoids, podoverines B–C, were isolated from the dried roots and rhizomes of *Sinopodophyllum emodi* using a Sephadex LH-20 column (SLHC) and high-speed counter-current chromatography (HSCCC). The 95% ethanol extract was partitioned with ethyl acetate in water. Target compounds from the ethyl acetate fraction were further enriched and purified by the combined application of SLHC and HSCCC. *n*-Hexane–ethyl acetate–methanol–water (3.5:5:3.5:5, *v*/*v*) was chosen as the two phase solvent system. The flow rate of mobile phase was optimized at 2.0 mL·min^−1^. Finally, under optimized conditions, 13.8 mg of podoverine B and 16.2 mg of podoverine C were obtained from 200 mg of the enriched sample. The purities of podoverines B and C were 98.62% and 99.05%, respectively, as determined by HPLC. For the first time, podoverins B and C were found in the genus *Sinopodophyllum*. Their structures were determined by spectroscopic methods (HR-ESI-MS, ^1^H-NMR, ^13^C-NMR, HSQC, HMBC). Their absolute configurations were elucidated by comparison of their experimental and calculated ECD spectra. The cytotoxic activities were evaluated against MCF-7 and HepG2 cell lines. The separation procedures proved to be practical and economical, especially for trace prenylated biflavonoids from traditional Chinese medicine.

## 1. Introduction

**Figure 1:**
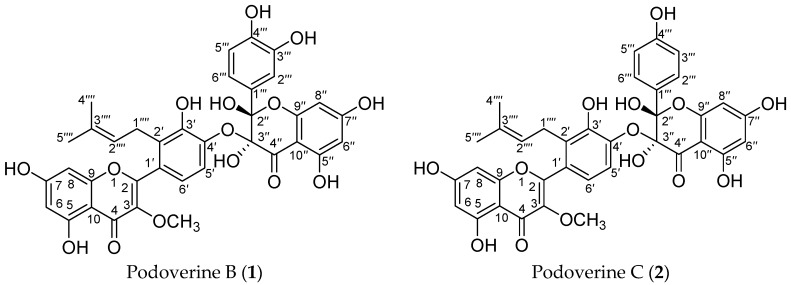
The chemical structures of two prenylated biflavonoids from *S. emodi.*

*Sinopodophyllum emodi* (Wall.) Ying, which belongs to the family of Berberidaceae, is a herbaceous perennial plant widely distributed in the Southwest of China [1]. Listed in Chinese Pharmacopoeia, the dried fruits are clinically applied to the treatment of amenorrhea, dead fetus, and placental retaining as a traditional Tibetan medicine [2]. Its roots and rhizomes have been used for the treatment of certain cancer, various verrucosis [1], constipation, parasitosis [3], rheumatoid ache [4], and pyogenic infection of skin tissue [5]. Previous chemical investigations on *S. emodi* revealed the presence of bioactive aryltetralin [1,3,4,5,6,7,8] and tetrahydrofuranoid lignans [9], flavonoids [2,10,11,12], steroids [13], and phenolics [14]. Although flavonoids have been the research focus of this plant [2,10,11,12], the biflavonoids, podoverines B, C (Figure 1), were found firstly in the genus *Sinopodophyllum*. As anti-inflammatory agents, podoverines B and C have been isolated from *Podophyllum versipelle* callus cell culture [15], further studies are not reported up to now. Naturally-occurring biflavonoids have exhibited a broad spectrum of biological activities, including anticancer, antibacterial, antifungal, antiviral, anti-inflammatory, analgesic, antioxidant, vasorelaxant, anticlotting, *etc.* [15,16]. As potential therapeutic drugs against cancer, biflavonoids strongly interfere in related pathways of the cancer cell growth and death while have little effect on normal cell proliferation [16]. Therefore, it is critical and urgently needed to develop a rapid and efficient method for the preparative isolation of podoverines B and C from *S. emodi*.

HSCCC is a support-free liquid-liquid chromatographic technology, based on partitioning of target compounds between two immiscible liquid phases. Some complications arising from solid absorbents can be eliminated, such as irreversible adsorption and denaturation of target compounds [17]. Compared with traditional liquid–solid chromatographic methods, it also has a large number of advantages, including high sample recovery, large loading capacity, low solvent consumption, acceptable efficiency, low cost, and the ease of scaling-up [18,19]. HSCCC has been widely used in the large scale preparative isolation and purification of various kinds of natural products. Although HSCCC has been developed for the purification of normal biflavonoid [20,21], there are no reports on the application of HSCCC for the preparative isolation of prenylated biflavonoids from natural sources. In this work, the HSCCC has been applied in combination with SLHC for the purification of two prenylated biflavonoids (podoverines B and C) from the roots and rhizomes of *S. emodi*. The HSCCC isolation conditions, including two-phase solvent system, flow rate, and revolution speed, were optimized. The chemical structures of the two target compounds were elucidated by HR-ESI-MS, ^1^H-NMR, ^13^C-NMR, HSQC, and HMBC. Their absolute configurations were determined by comparison of their experimental and calculated ECD spectra. The cytotoxic activities were also evaluated against MCF-7 and PC-3 cell lines by the MTT method. As far as we know, the HSCCC separation, absolute configurations, and cytotoxic activities of podoverines B and C are now reported for the first time.

## 2. Results and Discussion

### 2.1. Enrichment by Sephadex LH-20 Column

The roots and rhizomes of *S. emodi* is particularly rich in aryltetralin lactone lignans and normal flavonoids [22]. Based on the HPLC analysis, it was almost impossible for podoverines B and C to be detected in the ethyl acetate fraction. Furthermore, the polarities and molecular weights of prenylated biflavonoids were similar to those of aryltetralin lactone lignan glycosides. To enrich effectively the target compounds, SLHC chromatography was employed for pre-separation, eluting with a methanol–water gradient. The chromatographic parameters, including mobile phase composition, loading amount, and flow rate, were investigated to produce optimum separation efficiency.

**Figure 2:**
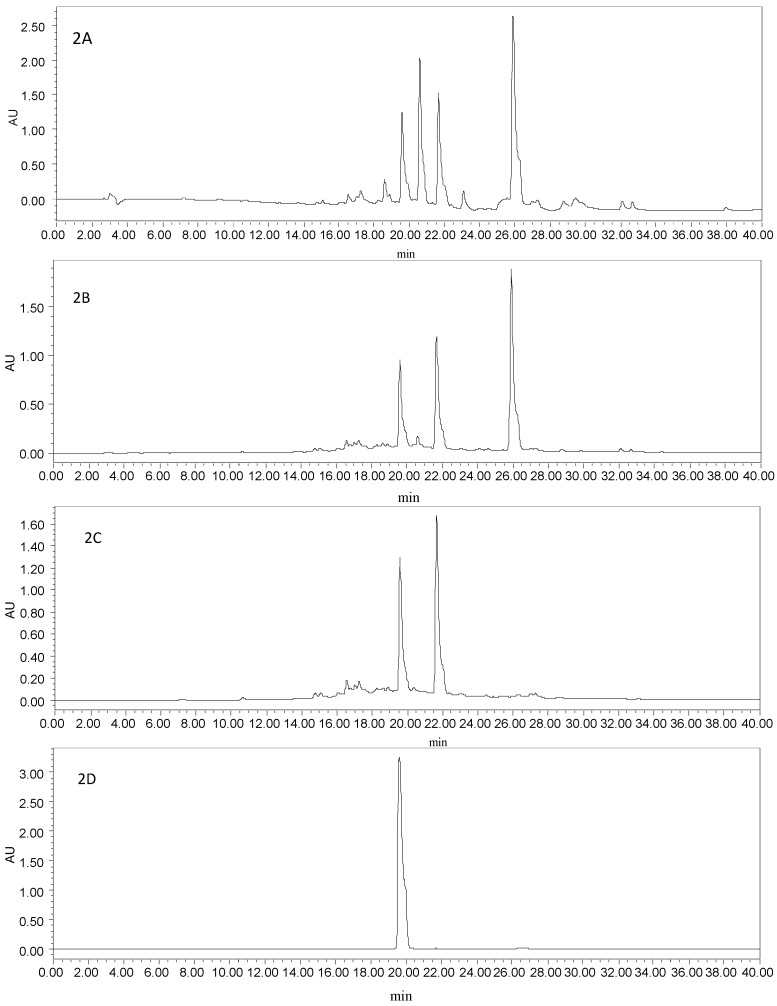

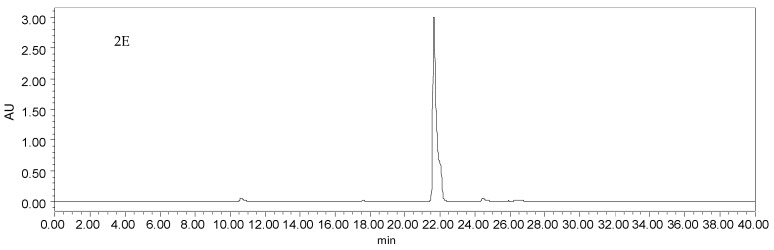
(**A**) HPLC chromatogram of the enriched sample from SLHC, which were isolated with the mixed solvents of dichloromethane–methanol; (**B**) HPLC chromatogram of the enriched sample from SLHC, which were isolated with isocratic 60% methanol; (**C**) HPLC chromatogram of the enriched sample from SLHC, which were isolated with a gradient methanol–water; (**D**) HPLC chromatogram of HSCCC peak fraction 1 in Figure 3; (**E**) HPLC chromatogram of HSCCC peak fraction 2 in Figure 3; Experimental conditions: column, a YMC-Pack ODS A column (5 μm, 250 mm × 4.6 mm); mobile phase, methanol (C) and 0.1% trifluoroacetic acid (D) at the gradient (20%–65% C at 0–20 min, 65%–100% C at 20–40 min); flow rate, 1.0 mL·min^−1^; detection wavelength, 254 nm; column temperature, 35 °C.

Two kinds of binary solvent systems were examined, including gradient dichloromethane–methanol and methanol–water. When the mixed solvent system of dichloromethane–methanol (*v*/*v*, 1:1, 1:1.5, 1:2, 0:1) was used as mobile phase, the target compounds were co-eluted, together with a lot of impurities with similar properties (Figure 2A). The different ratios of isocratic methanol (A)–water (B) (*v*/*v*, 10%, 20%, 30%, 40%, 50%, 60%, 70%, 100% A) were also investigated systematically. Brown pigments and lignans, not the target compounds, could be eluted by 10%–50% A. Two target compounds, together with another unknown constituent, mainly existed in 60% A (Figure 2B). Therefore, the target compounds were enriched in a gradient elution mode (50%, 60% A, Figure 2C).

**Figure 3:**
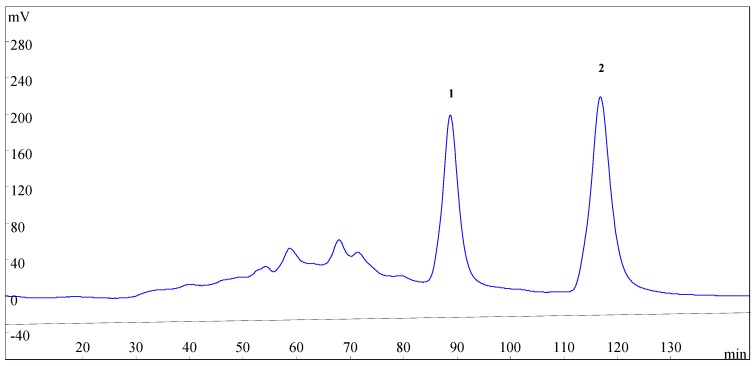
HSCCC chromatogram of the enriched sample from SLHC. Two-phase solvent system: *n*-hexane–ethyl acetate–methanol–water (3.5:5:3.5:5, *v*/*v*); mobile phase: the lower phase; stationary phase: the upper phase; flow rate: 2.0 mL·min^−1^; revolution speed: 800 rpm; detection wavelength: 254 nm; sample size: 200 mg enriched sample was dissolved in the solvent mixture of *n*-hexane–ethyl acetate–methanol–water (5 mL for each phase).

As sample loading amount exceeded column loading capacity, the separation efficiency on the SLHC decreased obviously. When it was more than 20 mg·g^−1^, a great deal of lignan glucosides were detected in the eluates containing target compounds. The separation of target compounds was satisfactory with sample loading amount from 10 to 20 mg·g^−1^. With the decrease of required Sephadex LH-20, the contents of target compounds increased in the eluates. According to these results, the optimal loading amount on the Sephadex LH-20 column was determined as 20 mg·g^−1^.

For SLHC, flow rate is also an important influencing factor. Generally, the lower the flow rate, the better the resolution. However, the lower the flow rate, the longer the elution time. The effect of flow rate on separation efficiency was further investigated. The contents of impurities in the collected fraction increased with flow rate increasing from 1 to 3 mL·min^−1^. The separation efficiency gradually increased with the flow rate decreasing from 1 to 0.6 mL·min^−1^. In order to reduce the total elution time, an ideal flow rate was selected at 1 mL·min^−1^.

### 2.2. Selection of Two-Phase Solvent System and Other Conditions of HSCCC

In HSCCC, a suitable two-phase solvent system was critical for successful separation. The two-phase solvent system was selected according to the following requirements [23]: (i) the target compounds should be stable and soluble in the selected system; (ii) the settling time of the two-phase solvent system should be shorter than 30 s; and (iii) the optimal volume ratio of the two-phase solvent system provides an ideal range of the coefficients (K, *i.e.*, usually between 0.5 and 2.5) for the target compounds. Small K values lead to the disappearance of peak resolution, while large K values tend to require a relatively large quantity of organic solvents, long operation time, and produce sample band broadening. For all of the selected two-phase solvent systems, the settling time was less than 20 s. In order to achieve ideal resolution of target compounds, the K values for *n*-hexane–ethyl acetate–methanol–water at different volume ratio were measured systematically (Table 1). Started with *n*-hexane–ethyl acetate–methanol–water (5:5:5:5 *v*/*v*), two target compounds were eluted close to each other near the solvent front. The distribution capacity of target compounds in the upper layer was adjusted by volume ratio of *n*-hexane–methanol or *n*-hexane–ethyl acetate in mixed solvent system. For the solvent systems consisted of *n*-hexane–ethyl acetate–methanol–water (2:5:2:5), two target compounds were retained in the column for a relatively long time (5 h). In those systems of *n*-hexane–ethyl acetate–methanol–water (4.5:5:4.5:5, and 6:4:5:5), *K* values were too small. Whereas in those systems of *n*-hexane–ethyl acetate–methanol–water (1:5:1:5), *K* values were too big. For *n*-hexane–ethyl acetate–methanol–water (4:5:4:5, 3.5:5:3.5:5 and 3:5:3:5), the *K* values were between 0.5 and 2.5. In contrast, with *n*-hexane–ethyl acetate–methanol–water at a ratio of 3.5:5:3.5:5, the target compounds were separated satisfactorily with high resolution (α = 1.25) in an acceptable run time. Thus, this solvent system was selected for subsequent HSCCC separation.

**Table 1:** The partition coefficients (*K*) and separation factors (α) of the target compounds in several solvent systems.

Solvent System	Ratio	*K* Value		α
		1	2	
*n*-Hexane–ethyl acetate–methanol–water	6:4:5:5	0.12	0.15	1.25
*n*-Hexane–ethyl acetate–methanol–water	5:5:5:5	0.18	0.23	1.28
*n*-Hexane–ethyl acetate–methanol–water	4.5:5:4.5:5	0.29	0.37	1.28
*n*-Hexane–ethyl acetate–methanol–water	4:5:4:5	0.60	0.69	1.15
*n*-Hexane–ethyl acetate–methanol–water	3.5:5:3.5:5	0.84	1.05	1.25
*n*-Hexane–ethyl acetate–methanol–water	3:5:3:5	1.41	1.64	1.16
*n*-Hexane–ethyl acetate–methanol–water	2:5:2:5	2.56	2.83	1.11
*n*-Hexane–ethyl acetate–methanol–water	1:5:1:5	5.93	6.14	1.04

The retention of the stationary phase is also one of the most important parameters in HSCCC. The retention of the stationary phase is highly correlated with the flow rate of the mobile phase and the revolution speed of the separation column [21]. Different flow rate (1.6, 1.8, 2.0, 2.2, and 2.5 mL·min^−1^) of the mobile phase and different revolution speed (600, 700, 800, 900 rpm) were investigated with the selected solvent system. At the flow rate of 2.2 and 2.5 mL·min^−1^, podoverine C (**2**) was separated satisfactorily, however, podoverine B (**1**) peak always coexisted with some nearby impurities. At the flow rate of 1.6, 1.8, and 2.0 mL·min^−1^, podoverine B (**1**) was separated from impurities in a higher resolution. The higher the retention of the stationary phase, the better the peak resolution. Low flow rate and high revolution speed increase the retention of the stationary phase [24]. However, with the decrease of the flow rate, the more elution time and more mobile phase will be needed, and the chromatographic peak is widened. High revolution speed may also produce broadening sample bands by violent pulsation [25] and reduce the life of instrument [26]. The retention of the stationary phase was poor at a revolution speed of 600 and 700 rpm, whereas the satisfactory retention was obtained at 800 rpm. Considering these aspects, a flow rate of 2.0 mL·min^−1^ and a revolution speed of 800 rpm were finally selected for HSCCC separation.

Under the optimized conditions, podoverines B (13.8 mg) and C (16.2 mg) were successfully obtained from 200 mg of the enriched sample in one-step elution and less than 150 min. Their purities were 98.62% and 99.05%, respectively. The retention percentage of the stationary phase was 62%.

### 2.3. Optimization of HPLC Conditions

As shown in Figure 2C, a considerable level of impurities, which showed similar polarities to two target compounds, were detected in the enriched sample. The only structural difference between two target compounds was that podoverine B has one more hydroxyl group at C-3′′′, so that their properties such as UV absorbance and chromatographic pattern were similar. The contents of podoverines B and C were 25.7% and 33.1%, based on the ratio of peak area, respectively. Optimal HPLC analytical conditions were required to ensure the baseline separation and accurate purity results of the target compounds. Thus, different elution mode, flow rate, column temperature, and detection wavelength were evaluated. For natural products with hydroxyl groups, acid is generally used for reducing the tailing and broadening of peak. To improve separation resolution, trifluoroacetic acid was added into the mobile phase. When isocratic methanol (C)–0.1% trifluoroacetic acid (*v*/*v*) (D) (85% C) was used as a mobile phase, the retention time of two target compounds were identical in the HPLC chromatogram. By decreasing methanol ratio in the mobile phase, two target compounds were separated gradually from each other, however, their retention times became longer and nearby impurities were almost hidden by the peak of podoverin B. The screening results indicated the gradient elution of methanol (C)–0.1% trifluoroacetic acid (*v*/*v*) (D) (20%–65% C at 0–20 min, 65%–100% C at 20–40 min) gave a satisfactory separation of the target compounds, when the flow rate, column temperature and detection wavelength were at 1.0 mL·min^−1^, 35 °C and 254 nm.

### 2.4. Identification of the Separated Peaks

The chemical structures of two prenylated biflavonoids were determined on the basis of spectroscopic evidences (HR-ESI-MS, ^1^H-NMR, ^13^C-NMR, HSQC, and HMBC in Supplementary Materials), and their absolute configurations were elucidated by CD analysis. The data were given in Table 2.

**Figure 4:**
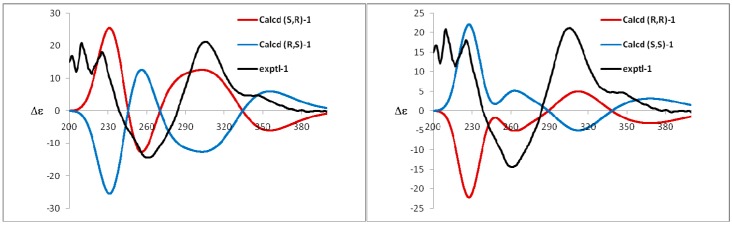
Experimental and calculated ECD spectra of compound **1**.

Compound **1** was obtained as a yellow amorphous powder and possessed a molecular formula C_36_H_30_O_15_ with twenty-two degrees of unsaturation, as revealed from its HR-ESI-MS analysis (*m*/*z* 685.1538 [M + H − H_2_O]^+^, calcd for C_36_H_29_O_14_, 685.1557; *m*/*z* 707.1355 [M + Na − H_2_O]^+^, calcd for C_36_H_28_O_14_Na, 707.1377; *m*/*z* 723.1093 [M + K − H_2_O]^+^, calcd for C_36_H_28_O_14_K, 723.1116). The ^1^H-NMR spectrum showed four aromatic systems including one 1,2,3,4-tetra-substituted benzene ring δ 7.13 (1H, d, *J* = 8.4 Hz), 7.01 (1H, d, *J* = 8.4 Hz), one 1,3,4-tri-substituted benzene ring δ 7.11 (1H, d, *J* = 2.1 Hz), 6.89 (1H, dd, *J* = 8.4, 2.1 Hz), 6.68 (1H, d, *J* = 8.4 Hz), and two 1,2,3,5-tetra-substituted benzene ring at *δ* 6.34 (1H, d, *J* = 1.6 Hz), 6.22 (1H, d, *J* = 1.6 Hz), and δ 5.97 (2H, s); One 3-methyl-2-butenyl δ 5.01 (1H, t, *J* = 7.0 Hz), 3.28 (2H, d, *J* = 7.0 Hz), 1.48 (3H, s), and 1.27 (3H, s); One aromatic methoxy group δ 3.67 (3H, s); One chelated phenolic hydroxyl group δ 12.55 (1H, s, 5-OH). The ^13^C-NMR and HSQC spectrum revealed one 3-methyl-2-butenyl δ 25.5, 121.2, 131.7, 17.3, 25.3; one aromatic methoxy group δ 60.1; one biflavone skeleton including two carbonyl group at δ 187.3, 178.0; four benzene rings, two oxygen-bearing olefinic carbons δ 139.0, 157.5; two di-oxygen-bearing aliphatic quaternary carbons δ 100.1, 90.2. The HMBC correlations of the aromatic protons δ 7.13 (1H, d, *J* = 8.4 Hz, H-6′) with C-2 (δ 157.5), the methylene group protons δ 3.28 (2H, d, *J* = 7.0 Hz) with C-2′ (δ 129.3), and the methoxy group protons δ 3.67 (3H, s) with C-3 (δ 139.0), in combination with one 1,2,3,5-tetra-substituted benzene ring at δ 6.34 (1H, d, *J* = 1.6 Hz), 6.22 (1H, d, *J* = 1.6 Hz), indicated that compound **1** contained podoverine A [15] as a subunit. Another subunit was identified as 2,3,3,5,7,3′,4′-heptahydroxyflavanone by the HMBC cross peak of the aromatic protons δ 7.11 (1H, d, *J* = 2.1 Hz, H-2′″) and 6.89 (1H, dd, *J* = 8.4, 2.1 Hz, 6′″) with C-2″ (δ 100.1), one 1,2,3,5-penta-substituted benzene ring *δ* 5.97 (2H, s), and two di-oxygen-bearing aliphatic quaternary carbons δ 100.1, 90.2. A careful comparison of the ^13^C-NMR spectra of **1** with podoverine A indicated that the subunit of podoverine A was substituted at C-4′, which was confirmed by a chemical shift change from δ 138.3 (C-3′), 142.0 (C-4′), and 115.1 (C-5′) in **1** to δ 144.0 (C-3′), 147.3 (C-4′), and 113.2 (C-5′) in podoverine A. An ether bridge C-4′-O-C-3″ of the two flavonoid subunits was determined by one di-oxygen-bearing aliphatic quaternary carbons δ 90.2 (C-3″). Detailed elucidation on 1D and 2D NMR data led to the construction of the planar structure of **1**. Despite repeated experiments, suitable crystals of **1** for X-ray diffraction were not obtained successfully. The absolute configurations of C-2″ and C-3″ were extrapolated by comparing the experimental and calculated CD spectra, the latter performed by density functional theory. The results showed that experimental and calculated spectra for the 2″*S*, 3″*R*-isomer were in good agreement (Figure 4). Therefore, the absolute configuration at C-2″ and C-3″ were respectively deduced to be *S* and *R*. On the basis of the above evidences and related literature [15], compound **1** was determined as podoverine B.

**Table 2:** ^1^H-NMR and ^13^C-NMR spectroscopic data for compounds **1**–**2**.

Position	1 ^a^		2 ^a^	
	δ_C_	δ_H_	δ_C_	δ_H_
2	157.5		157.4	
3	139.0		139.0	
4	178.0		178.0	
5	161.4		161.3	
6	98.7	6.22 (1H, d, *J* = 1.6)	98.7	6.22 (1H, d, *J* = 2.1)
7	164.3		164.3	
8	93.7	6.34 (1H, d, *J* = 1.6)	93.7	6.34 (1H, d, *J* = 2.1)
9	156.8		156.8	
10	104.7		104.7	
1′	124.7		124.3	
2′	129.3		129.3	
3′	138.3		138.3	
4′	142.0		142.0	
5′	115.1	7.01 (1H, d, *J* = 8.4)	115.1	7.01 (1H, d, *J* = 8.4)
6′	124.1	7.13 (1H, d, *J* = 8.4)	124.1	7.12 (1H, d, *J* = 8.4)
2″	100.1		100.2	
3″	90.2		90.2	
4″	187.3		187.3	
5″	163.0		163.1	
6″	97.1	5.97 (1H, s)	97.1	5.98 (1H, d, *J* = 2.1)
7″	167.8		167.8	
8″	96.2	5.97 (1H, s)	96.1	5.99 (1H, d, *J* = 2.1)
9″	159.1		159.1	
10″	99.8		99.7	
1′′′	124.1		124.1	
2′′′	114.7	7.11 (1H, d, *J* = 2.1)	129.1	7.44 (1H, dd, *J* = 6.9, 2.1)
3′′′	144.6		114.7	6.75 (1H, dd, *J* = 6.9, 2.1)
4′′′	146.8		158.6	
5′′′	115.6	6.68 (1H, d, *J* = 8.4)	114.7	6.75 (1H, dd, *J* = 6.9, 2.1)
6′′′	119.1	6.89 (1H, dd, *J* = 8.4, 2.1)	129.	7.44 (1H, dd, *J* = 6.9, 2.1)
OCH_3_	60.1	3.67 (3H, s)	60.1	3.66 (3H, s)
1″″	25.5	3.28 (2H, d, *J* = 7.0)	25.5	3.27 (2H, d, *J* = 6.8)
2″″	121.2	5.01 (1H, t, *J* = 7.0)	121.2	5.01 (1H, t, *J* = 6.8)
3″″	131.7		131.6	
4″″	17.3	1.27 (3H, s)	17.3	1.26 (3H, s)
5″″	25.3	1.48 (3H, s)	25.2	1.48 (3H, s)
OH		12.55 (1H, s)		12.55 (1H, s)

^a^ NMR spectroscopic data were recorded in DMSO-*d*_6_ at 500 MHz (^1^H-NMR) and 125 MHz (^13^C-NMR).

Compound **2** was obtained as a yellow amorphous powder. Its ^1^H- and ^13^C-NMR were quite similar to those of **1**, except that one 1,4-di-substituted benzene ring δ 7.44 (2H, dd, *J* = 6.9, 2.1 Hz), 6.75 (2H, dd, *J* = 6.9, 2.1 Hz), were observed instead of one 1,3,4-tri-substituted benzene ring in **1**. Those were further supported by HR-ESI-MS, which gave a molecular formula C_36_H_30_O_14_ (*m*/*z* 669.1589 [M + H − H_2_O]^+^, calcd for C_36_H_29_O_13_, 669.1608; *m*/*z* 691.1409 [M + Na − H_2_O]^+^, calcd for C_36_H_28_O_13_Na, 691.1428; *m*/*z* 707.1173 [M + K − H_2_O]^+^, calcd for C_36_H_28_O_13_K, 707.1167), being 16 mass-units less than that of **1**, respectively. The CD spectrum of **2** was identical with **1** which has the absolute configuration (2″*S*, 3″*R*). Thus, compound **2** were identified as podoverine C [15].

### 2.5. The Cytotoxic Activity of Target Compounds

Target compounds were evaluated for their *in vitro* cytotoxic activity against MCF-7 and HepG2 cell lines using MTT assay [8], and IC_50_ values were summarized in Table 3. According to IC_50_ values, compound **1** showed higher cytotoxic activities than **2**, indicating that the hydration at C-3′ resulted in a greater increase of cytotoxic activity.

**Table 3:** Cytotoxic activity of target compounds (IC_50_, μM).

Compound	MCF-7	HepG2
**1**	29.8 ± 2.0	41.6 ± 1.9
**2**	42.6 ± 3.1	67.5 ± 2.6
etoposide	3.17 ± 0.25	0.48 ± 0.03

## 3. Experimental Section

### 3.1. Apparatus

The preparative HSCCC experiments were performed on a Model GS-10A high-speed counter-current chromatography (Beijing Institute of New Technology Application, Beijing, China). The instrument was equipped with a PTFE (polytetrafluoroethylene) multilayer coil column (i.d. of the tubing = 1.6 mm, a total capacity = 230 mL) and a manual sample injection valve with a 10 mL loop. The revolution radius was 5 cm, and the β value of the multilayer coil ranged from 0.5 at internal terminal to 0.8 at the external terminal. The maximum revolution speed could be controlled up to 1000 rpm by a speed controller. The HSCCC system was also equipped with BF-2002 CT11 signal collection cell (Chromatography Center of Beifenruili Group Company, Beijing, China), a Model NS-1007A constant-flow pump and a Model 8823B-UV detector (Beijing Institute of New Technology Application, Beijing, China) at 254 nm. The data were collected with HW-2000 chromatography workstation (Qianpu Software Co. Ltd., Shanghai, China).

Analytical HPLC was performed on high-performance liquid chromatography system with a Waters Alliance 2489 separations module equipped with a Waters 2695 UV/visible detector, a quaternary pump, a column temperature control module, and a Waters 717 plus autosampler (Milford, MA, USA). Empower pro data handling system (Waters Co., Milford, CT, USA) was employed to carry out data acquisition.

The structures of the target compounds were identified by high resolution electrospray ionization mass (HR-ESI-MS) spectrometer (Shimadzu LC-MS 2010, Japan) and nuclear magnetic resonance (NMR) spectrometer (Bruker AM 500, Fällanden, Switzerland). CD spectra were measured on Bio-logic MOS 450 spectropolarimeter (Bio-logic Co., Claix, France). IR spectra were determined on a Nicolet is 10 Microscope Spectrometer (Thermo Scientific, San Jose, CA, USA). UV spectra were recorded on a UV-2401PC apparatus (Shimadzu Corporation, Kyoto, Japan).

### 3.2. Materials and Reagents

All organic solvents for sample preparation and HSCCC separation were of analytical grade (Fuyu Chemical Reagent Co. Ltd., Tianjin, China). Methanol for HPLC analysis was of chromatographic grade (Siyou Biology Medical Tech Co. Ltd., Tianjin, China), and water was purified by means of a water purifier (18.2 MΩ) (Wanjie Water Treatment Equipment Co. Ltd., Hangzhou, China). The target compounds were enriched by Sephadex LH-20 (Amersham Pharmacia Biotech AB, Uppsala, Sweden). Biological reagents were obtained from Sigma Company (St. Louis, MO, USA). Human heptocellular (HepG2) and breast (MCF-7) cell lines were from Institute of Materia Medica, Chinese Academy of Medical Sciences and Peking Union Medical College (Beijing, China).

The plant materials were collected in Deqin, Yunnan province, People’s Republic of China, in September 2014, and were identified as the roots and rhizomes of *S. emodi* (Wall.) Ying according to Chinese Traditional Medicine Dictionary by Professor Cheng-ming Dong (School of Pharmacy, Henan University of Traditional Chinese Medicine).

### 3.3. Preparation of the Crude Extract

The dried roots and rhizomes of *S. emodi* were ground to powder by a disintegrator. The powders (3.0 kg) were extracted under reflux by 10-fold amounts of 95% ethanol. The extraction procedure was then repeated twice. The extracts were combined together, filtrated with cotton, and then concentrated under reduced pressure to give brown syrup (639 g). This syrup was suspended in 3 L distilled water, and then partitioned with equal volumes of ethyl acetate. After concentration and freeze-drying, the ethyl acetate fraction (25 g) were stored at −10 °C for subsequent experiments.

### 3.4. Encrichment of the Target Compounds by Sephadex LH-20 Column

The ethyl acetate fraction (6.6 g) was dissolved ultrasonically in methanol (10 mL), and filtered by 0.45 μm microporous membrane. The SLHC was packed as follows: The exit of the chromatographic column (140 cm length × 4 cm i.d.) was plugged with glass wool to retain solids. Sephadex LH-20 (330 g) was swollen with methanol for 4 h. The swollen Sephadex LH-20 slurry was poured into the column in a continuous motion. The column was rinsed with methanol. Before applying the sample, the column was equilibrated with 2 L of 50% methanol, and the level was lowered to the stationary phase. The filtrate containing target compounds was loaded onto the column, and the elution was run with a methanol (A)–water (B) gradient (50%, 60% A, each 2000 mL) at a constant flow rate of 1 mL·min^−1^. According to TLC results, the eluates containing target compounds were collected and concentrated under reduced pressure. The enrichment procedure was then repeated twice. The enriched sample was stored at −10 °C for the subsequent HSCCC separation.

### 3.5. Further Purification by HSCCC

#### 3.5.1. Determination of the Partition Coefficient (K) Value

By HPLC, the partition coefficients (*K*) of target compounds were determined as follows: 10 mg of the enriched sample and 2 mL of the each phase of equilibrated two-phase solvent system were added into a 10 mL centrifuge tube. To achieve the thorough equilibration of target compounds between the two phases, the centrifuge tube was then stoppered, vortexed for 1 min, and kept for 30 min at room temperature. The upper and lower phases were separated and evaporated to dryness under N_2_ gas. The residue of each phase was re-dissolved in methanol. Then an aliquot of each phase (20 μL) was subjected to HPLC analysis. The *K* value was expressed as the ratio of the peak area of a given compound in the upper phase divided by that in the lower phase.

#### 3.5.2. Preparation of Two-Phase Solvent System and Sample Solution

Two-phase solvent system for HSCCC was prepared by mixing the corresponding solvents in a separatory funnel at room temperature and thoroughly equilibrated for more than 12 h. Then the lower phase and upper phase were separated shortly and degassed by sonication for 30 min before use. The sample solution was prepared by dissolving 200 mg enriched sample in the solvent mixture of *n*-hexane–ethyl acetate–methanol–water (5 mL for each phase).

#### 3.5.3. HSCCC Separation Procedure

HSCCC separation was performed as follows: the multilayer coiled column was entirely filled with the upper stationary phase of the solvent system. Then, the apparatus was run at a revolution speed of 800 rpm. In the meantime, the lower mobile phase was pumped into the column in a head-to-tail mode at a flow rate of 2.0 mL·min^−1^. After hydrodynamic equilibrium was established in the column, as indicated by the lower mobile phase front emerging from the tail outlet, about 10 mL of the enriched sample solution was introduced into the column through the injection valve. The eluates from the column outlet were continuously monitored by a UV detector at 254 nm. The fractions during 84–95 min (peak 1) and 112–125 min (peak 2) were collected respectively according to the obtained chromatographic profile (Figure 3). Each collection was evaporated under N_2_ gas. The purified compounds were stored at −20 °C before subsequent purity and NMR analyses. After the separation experiment, all solvents in the HSCCC column were ejected with N_2_ gas, and the retention of stationary phase was computed. All of the experiments were performed at room temperature (25 °C).

#### 3.5.4. HPLC Analysis and Identification of HSCCC Peaks

The enriched sample from SLHC and each purified HSCCC peak were analyzed on a YMC-Pack ODS A column (5 μm, 250 mm × 4.6 mm) at 35 °C (Figure 2C–E). A methanol (C)–0.1% trifluoroacetic acid (*v*/*v*) (D) system was used as the mobile phase in gradient elution mode as follows: 20%–65% C at 0–20 min, 65%–100% C at 20–40 min. The flow rate of the mobile phase was 1.0 mL·min^−1^. The eluates were monitored at 254 nm by a UV-VIS detector. The sample concentration is 0.5 mg·mL^−1^ for the HSCCC peaks, and 1.5 mg·mL^−1^ for the enriched sample from *S. emodi*. All samples were injected with the volume of 20 μL. Based on the peak area normalized to the sum of all observed HPLC peak areas, the purities of the isolated biflavonoids were determined.

*Podoverine B* (**1**). yellow, amorphous powder; ${\left[\alpha \right]}_{\text{D}}^{25}$ 201.5 (*c* 0.20, MeOH); CD (MeOH) λ_max_ (∆ε) 228 (+15.9), 262 (−13.2), 296 (+16.2) nm; UV (MeOH) λ_max_ (log ε) 261 (3.01), 304 (2.92), 338 (1.85) nm; IR (neat) ν_max_ 3301, 2969, 2928, 1637, 1608, 1591, 1507, 1473, 1438, 1357, 1266, 1155, 1087 cm^−1^; HR-ESI-MS (positive): *m*/*z* 685.1538 [M + H − H_2_O]^+^ (calcd for C_36_H_29_O_14_, 685.1557), *m*/*z* 707.1355 [M + Na − H_2_O]^+^ (calcd for C_36_H_28_O_14_Na, 707.1377), *m*/*z* 723.1093 [M + K − H_2_O]^+^ (calcd for C_36_H_28_O_14_K, 723.1116); NMR data (DMSO-*d*_6_), see Table 2.

*Podoverine C* (**2**). yellow, amorphous powder; ${\left[\alpha \right]}_{\text{D}}^{25}$ 204.8 (*c* 0.26, MeOH); CD (MeOH) λ_max_ (∆ε) 228 (+14.6), 262 (−12.5), 295 (+15.8) nm; UV (MeOH) λ_max_ (log ε) 260 (0.14), 302 (0.11), 337 (0.09) nm; IR (neat) ν_max_ 3233, 2970, 2929, 1638, 1608, 1591, 1508, 1473, 1440, 1358, 1264, 1156, 1086 cm^−1^; HR-ESI-MS (positive): *m*/*z* 669.1589 [M + H − H_2_O]^+^ (calcd for C_36_H_29_O_13_, 669.1608), *m*/*z* 691.1409 [M + Na − H_2_O]^+^ (calcd for C_36_H_28_O_13_Na, 691.1428), *m*/*z* 707.1173 [M + K − H_2_O]^+^ (calcd for C_36_H_28_O_13_K, 707.1167); NMR data (DMSO-*d*_6_), see Table 2.

#### 3.5.5. Computational Methods

The CONFLEX [27,28] searches based on molecular mechanics with MMFF94S force fields were performed for (SR)-**1** and (RR)-**1**, which gave four stable conformers, respectively. Selected those conformers were further optimized by the density functional theory method at the B3LYP/6-31G (d) level in Gaussian 09 program package [29], which was further checked by frequency calculation and resulted in no imaginary frequencies. The ECD of the conformers of **1** was then calculated by the TDDFT method at the B3LYP/6-31++G (d, p) level with the CPCM model in methanol solution. The calculated ECD spectra for each conformation were combined after Boltzman weighting according to their population contribution using SpecDis 162 software (Revision D.01, Gaussian Inc., Wallingford, CT, USA).

#### 3.5.6. *In Vitro* Cytotoxic Assays

Carcinoma cells were maintained in RPMI-1640 medium containing 10% heat-inactivated fetal bovine serum, penicillin (100 units/mL), and streptomycin (100 ug/mL) under humidified air with 5% CO_2_ at 37 °C. Exponentially growing cells were seeded into 96-well tissue culture-treated plates and pre-cultured for 24 h. The tested compounds at various concentrations were added, and the cells were incubated for additional 48 h. The cytotoxic activity was evaluated by MTT assay [8], and the IC_50_ values were obtained from dose-response curves.

## 4. Conclusions

*Sinopodophyllum emodi* is a well-known ethnic medicine with a long history. Previous chemical and pharmacological investigations indicated that flavonoids and lignans are mainly responsible for the biological activities of the plant. However, only thirty-six flavonoids had been isolated and identified, and their pharmacological properties were still a neglected field so far [2,10,11,12,30,31,32]. For the first time, two prenylated biflavonoids, podoverine B (**1**) and C (**2**), were extracted, isolated and identified from the genus *Sinopodophyllum*, and their cytotoxic activities were evaluated against MCF-7 and HepG2 cell lines. SLHC and HSCCC were developed respectively to enrich and purify two prenylated biflavonoids from *S. emodi*. By the developed method, 13.8 mg of podoverine B and 16.2 mg of podoverine C were obtained with the purities of over 98%. With multiple biological properties, biflavonoids are serving as a rich source of the health products and lead compounds for drug design. However, the biflavonoids generally coexist with other types of natural products or are trace in a complex biological organism. Furthermore, their structures are diverse and complex. For further bioactive investigation or quality control of related traditional Chinese medicine, it is crucial to develop the viable methods for separation and purification of trace natural products with complex structures, especially prenylated biflavonoids. Overall results of our study demonstrated that the combined application of SLHC and HSCCC would be a desirable separation pattern for trace prenylated biflavonoids from *S. emodi*.

## Supplementary Materials

Supplementary materials can be accessed at: http://www.mdpi.com/1420-3049/21/1/10/s1.
