# Supplementary material for: Preparative Isolation of Two Prenylated Biflavonoids from the Roots and Rhizomes of Sinopodophyllum emodi by Sephadex LH-20 Column and High-Speed Counter-Current Chromatography
Source: Molecules. 2015 Dec 23;21(1):10. doi: 10.3390/molecules21010010 (PMC6273534; doi:10.3390/molecules21010010)
Supplement: Supplementary file 1 [file molecules-21-00010-s001.pdf]

Supplementary Materials: Preparative Isolation of Two Prenylated Biflavonoids from the Roots and Rhizomes of *Sinopodophyllum emodi* by Sephadex LH-20 Column and High-Speed Counter-Current Chromatography

Yan-Jun Sun, Li-Xin Pei, Kai-Bo Wang, Yin-Shi Sun, Jun-Min Wang, Yan-Li Zhang, Mei-Ling Gao and Bao-Yu Ji

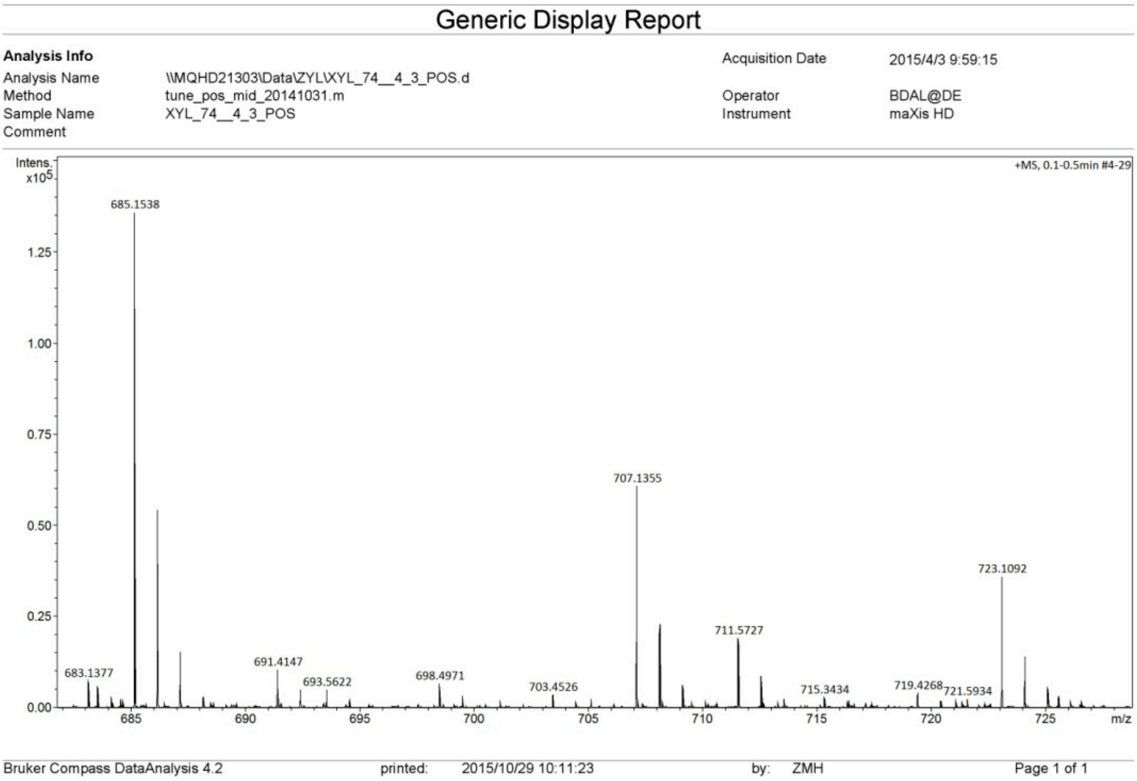

Figure S1. The HR-ESI-MS spectra of podoverine B (**1**).

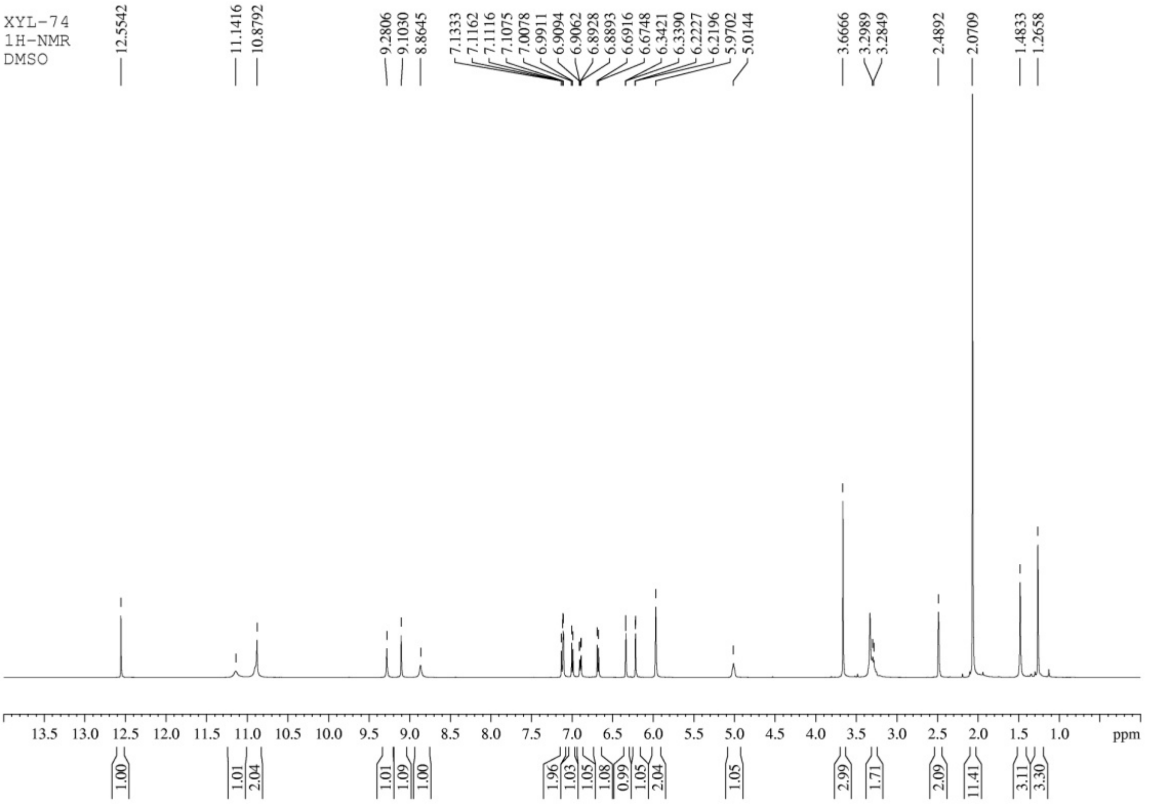

Figure S2. The ^{1}H-NMR spectra of podoverine B (**1**).

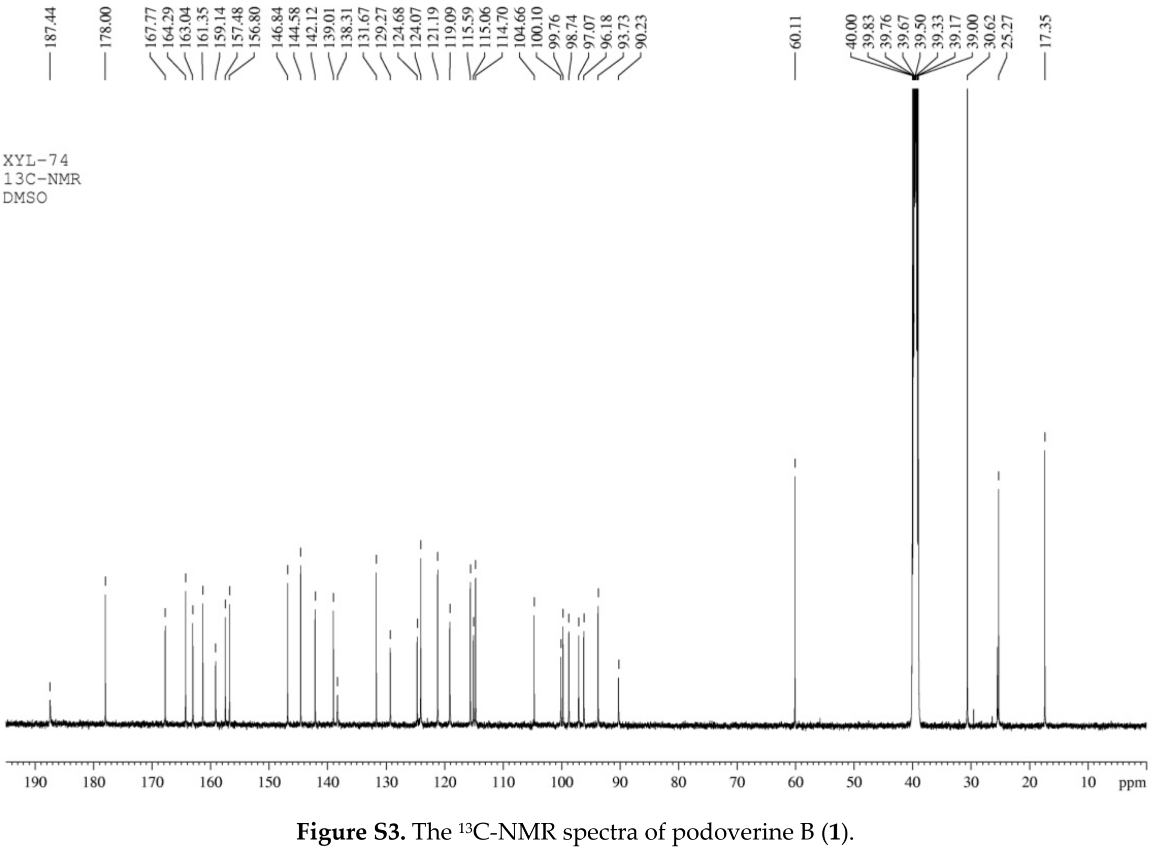

Figure S3. The ^{13}C-NMR spectra of podoverine B (**1**).

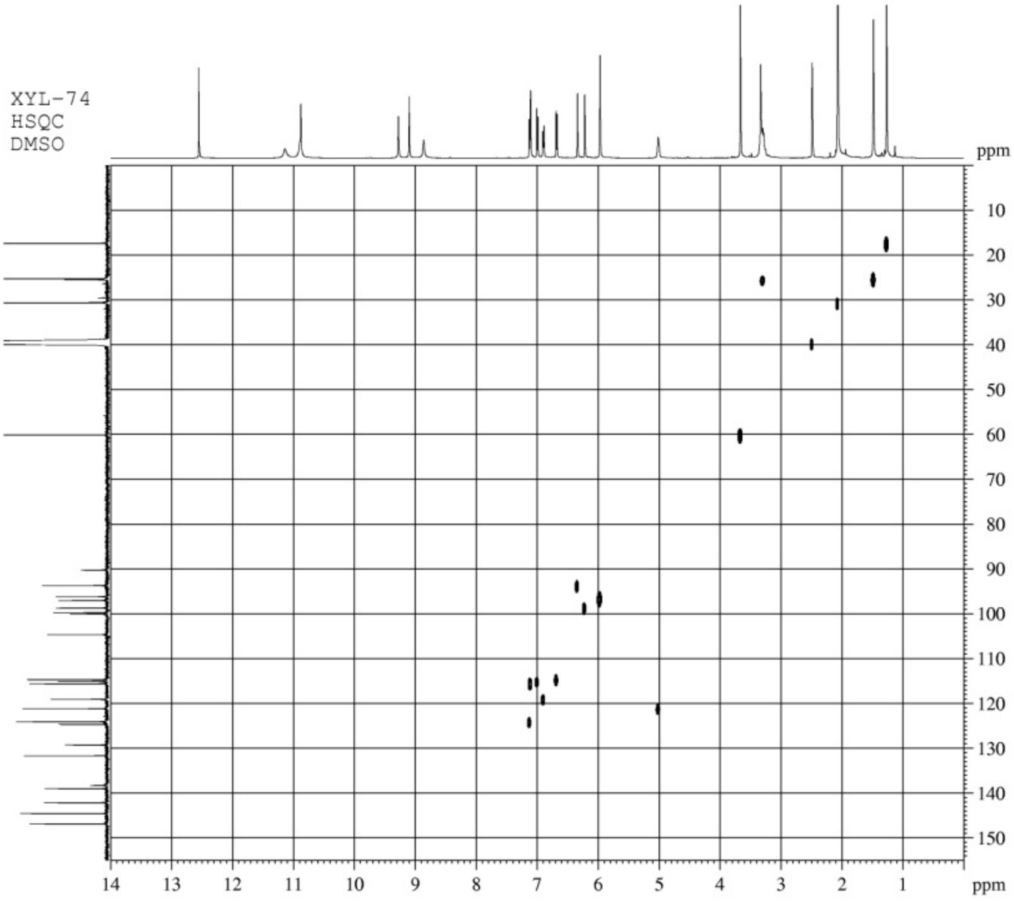

Figure S4. The HSQC spectra of podoverine B (**1**).

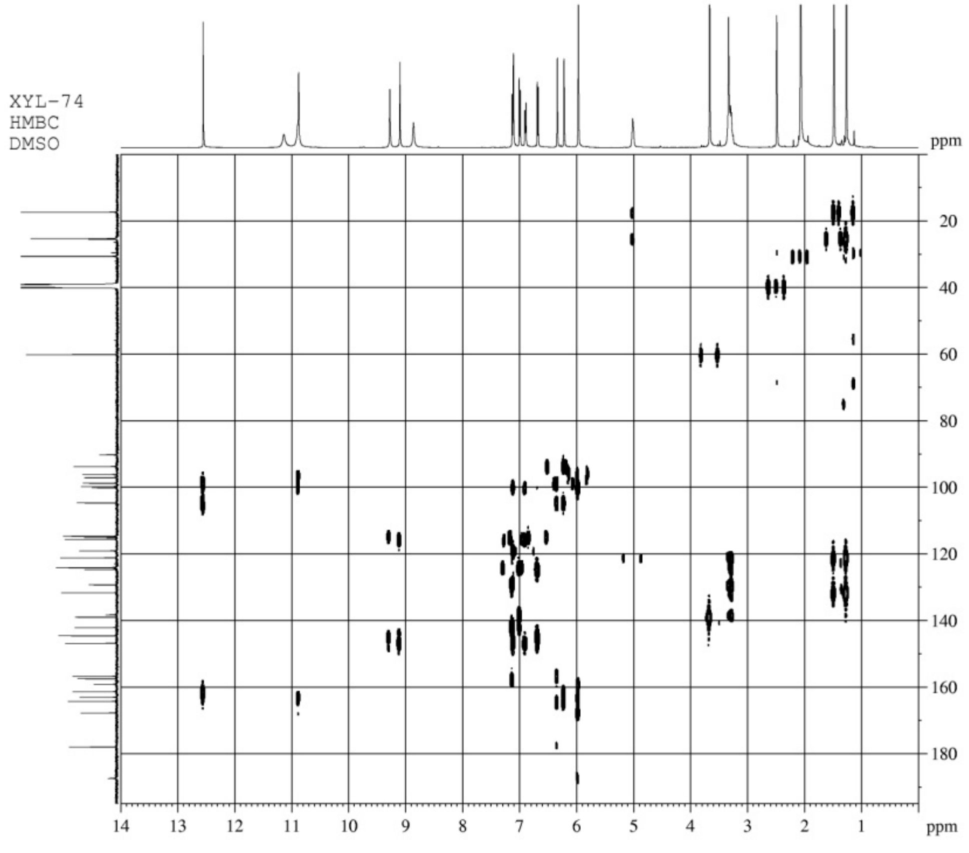

Figure S5. The HMBC spectra of podoverine B (**1**).

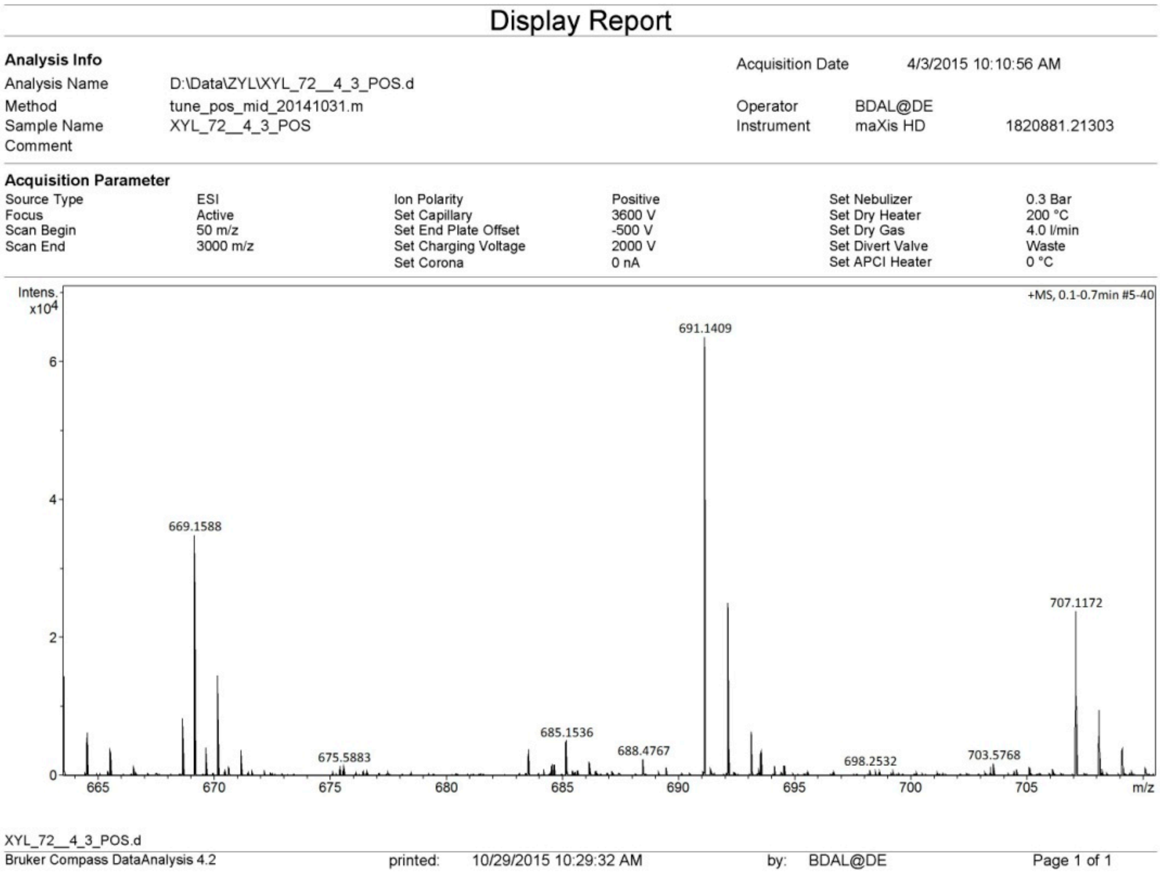

Figure S6. The HR-ESI-MS spectra of podoverine C (**2**).

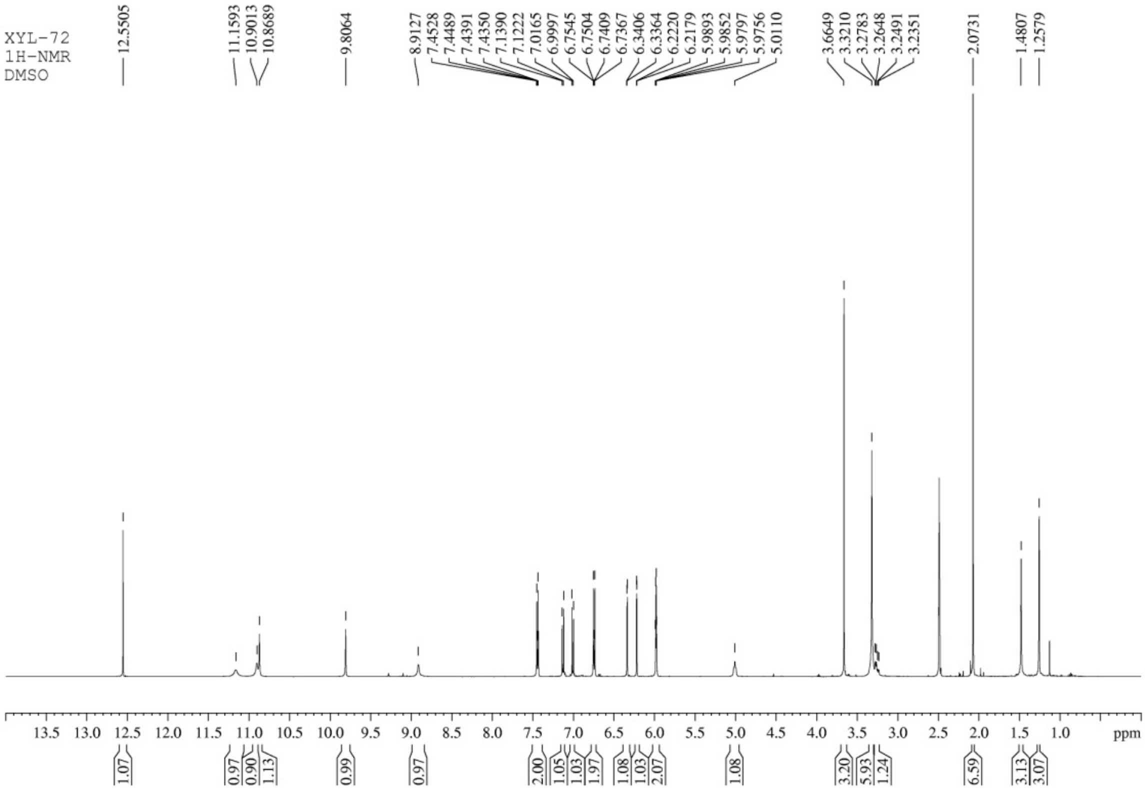

Figure S7. The ^{1}H-NMR spectra of podoverine C (**2**).

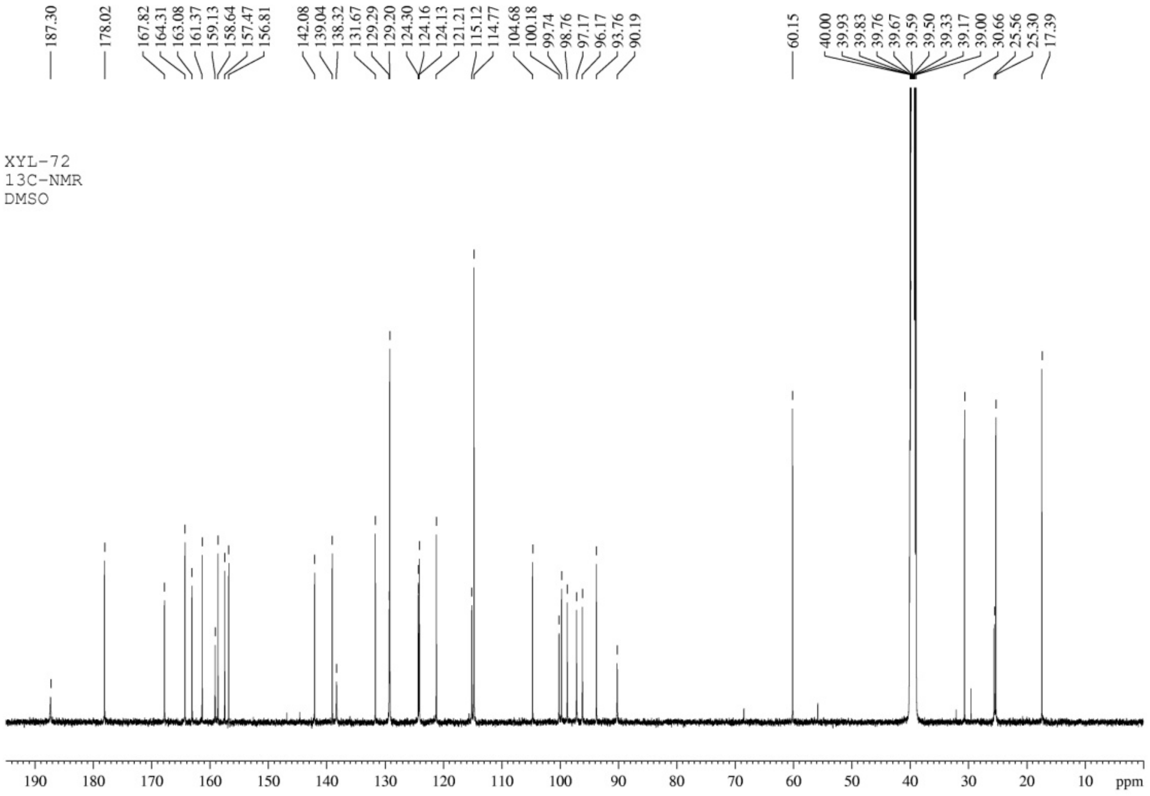

Figure S8. The ^{13}C-NMR spectra of podoverine C (**2**).

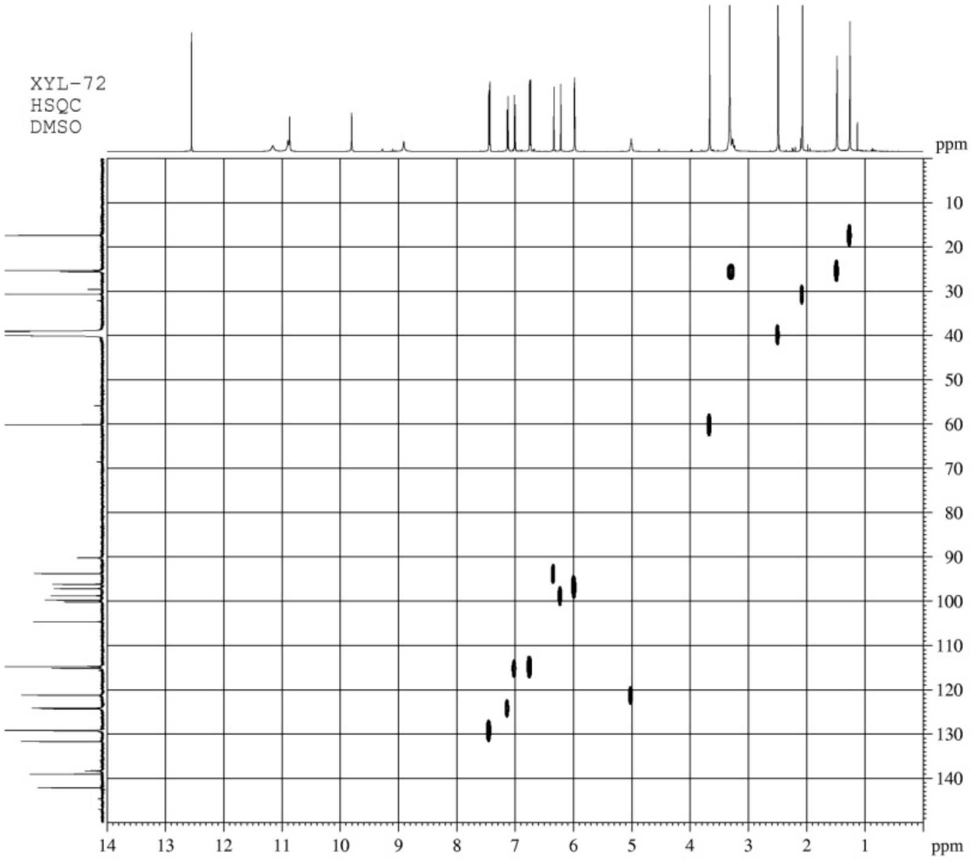

Figure S9. The HSQC spectra of podoverine C (**2**).

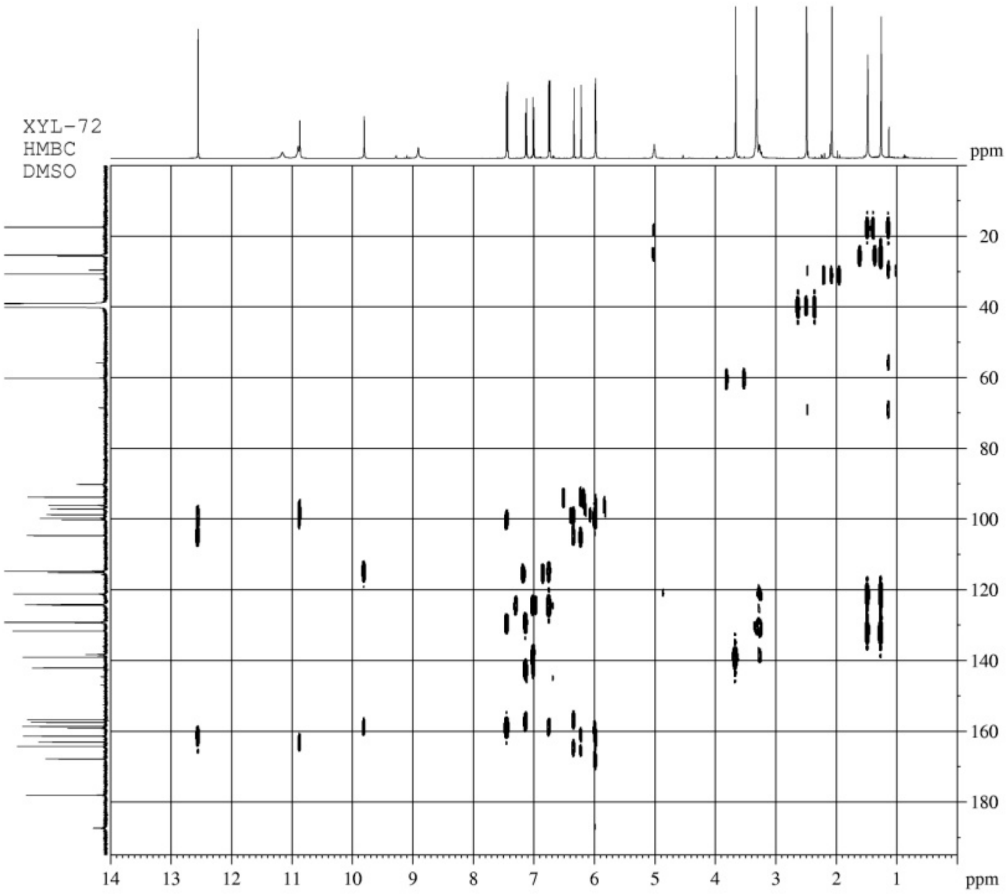

Figure S10. The HMBC spectra of podoverine C (**2**).
